# Complete genome sequence of *Meiothermus ruber* type strain (21^T^)

**DOI:** 10.4056/sigs.1032748

**Published:** 2010-07-29

**Authors:** Brian J Tindall, Johannes Sikorski, Susan Lucas, Eugene Goltsman, Alex Copeland, Tijana Glavina Del Rio, Matt Nolan, Hope Tice, Jan-Fang Cheng, Cliff Han, Sam Pitluck, Konstantinos Liolios, Natalia Ivanova, Konstantinos Mavromatis, Galina Ovchinnikova, Amrita Pati, Regine Fähnrich, Lynne Goodwin, Amy Chen, Krishna Palaniappan, Miriam Land, Loren Hauser, Yun-Juan Chang, Cynthia D. Jeffries, Manfred Rohde, Markus Göker, Tanja Woyke, James Bristow, Jonathan A. Eisen, Victor Markowitz, Philip Hugenholtz, Nikos C. Kyrpides, Hans-Peter Klenk, Alla Lapidus

**Affiliations:** 1 DSMZ - German Collection of Microorganisms and Cell Cultures GmbH, Braunschweig, Germany; 2 DOE Joint Genome Institute, Walnut Creek, California, USA; 3 Los Alamos National Laboratory, Bioscience Division, Los Alamos, New Mexico, USA; 4 Biological Data Management and Technology Center, Lawrence Berkeley National Laboratory, Berkeley, California, USA; 5 Oak Ridge National Laboratory, Oak Ridge, Tennessee, USA; 6 HZI – Helmholtz Centre for Infection Research, Braunschweig, Germany; 7 University of California Davis Genome Center, Davis, California, USA

**Keywords:** thermophilic, aerobic, non-motile, free-living, Gram-negative, *Thermales*, *Deinococci*, GEBA

## Abstract

*Meiothermus ruber* (Loginova *et al.* 1984) Nobre *et al*. 1996 is the type species of the genus *Meiothermus*. This thermophilic genus is of special interest, as its members share relatively low degrees of 16S rRNA gene sequence similarity and constitute a separate evolutionary lineage from members of the genus *Thermus*, from which they can generally be distinguished by their slightly lower temperature optima. The temperature related split is in accordance with the chemotaxonomic feature of the polar lipids. *M. ruber* is a representative of the low-temperature group. This is the first completed genome sequence of the genus *Meiothermus* and only the third genome sequence to be published from a member of the family *Thermaceae*. The 3,097,457 bp long genome with its 3,052 protein-coding and 53 RNA genes is a part of the *** G****enomic* *** E****ncyclopedia of* *** B****acteria and* *** A****rchaea * project.

## Introduction

Strain 21^T^ (= DSM 1279 = ATCC 35948 = VKM B-1258) is the type strain of the species *Meiothermus ruber*, which is the type species of the genus *Meiothermus* [1]. Strain 21^T^ was first described as a member of the genus *Thermus* by Loginova and Egorova in 1975 [2], but the species name to which it was assigned was not included on the Approved Lists of Bacterial Names [3]. Consequently *Thermus ruber* was revived, according to Rule 28a of the International Code of Nomenclature of Bacteria [4] in 1984 [5]. It received its current name in 1996 when transferred from the genus *Thermus* into the then novel genus *Meiothermus* by Nobre *et al*. [1]. Currently, there are eight species placed in the genus *Meiothermus* [6]. The genus name derives from the Greek words ‘meion’ and ‘thermos’ meaning ‘lesser’ and ‘hot’ to indicate an organism in a less hot place [1,6]. The species epithet derives from the Latin word ‘ruber’ meaning red, to indicate the red cell pigmentation [5,6]. Members of the genus *Meiothermus* were isolated from natural hot springs and artificial thermal environments [1] in Russia [5], Central France [7], both Northern and Central Portugal [8,9], North-Eastern China [10], Northern Taiwan [11] and Iceland [12]. Interestingly, the genus *Meiothermus* is heterogeneous with respect to pigmentation. The yellow pigmented species also form a distinct group on the basis of the 16S rRNA gene sequence similarity, with the red/orange pigmented strains forming two groups, one comprising *M. silvanus* and the other the remaining species [9,10]. Like all members of the *Deinococci* the lipid composition of the cell membrane of members of the genus *Meiothermus* is based on unusual and characteristic structures. Here we present a summary classification and a set of features for *M. ruber* 21^T^, together with the description of the complete genomic sequencing and annotation.

## Classification and features

The 16S rRNA genes of the seven other type strains in the genus *Meiothermus* share between 88.7% (*M. silvanus*) [13] and 98.8% (*M. taiwanensis*) [14] sequence identity with strain 21^T^, whereas the other type strains from the family *Thermaceae* share 84.5 to 87.6% sequence identity [15]. *Thermus* sp. R55-10 from the Great Artesian Basin of Australia (AF407749), as well as other reference strains, *e.g*. 16105 and 17106 [12], and the uncultured bacterial clone 53-ORF05 from an aerobic sequencing batch reactor (DQ376569) show full length 16S rRNA sequences identical to that of strain 21^T^. A rather large number of isolates with almost identical 16S rRNA gene sequences originates from the Great Artesian Basin of Australia, clone R03 (AF407684), and various hot springs in Hyogo, Japan (strain H328; AB442017), Liaoning Province, China (strain L462; EU418906, and others), Thailand (strain O1DQU (EU376397), a Finnish paper production facility (strain L-s-R2A-3B.2; AM229096) and others), but also the not validly published ‘*M. rosaceus*’ (99.9%) [16] from Tengchong hot spring in Yunnan (China). Environmental samples and metagenomic surveys do not surpass 81-82% sequence similarity to the 16S rRNA gene sequence of strain 21^T^, indicating a rather mixed impression about the environmental importance of strains belonging to the species *M. ruber*, as occurring only in very restricted extreme habitats (status August 2009).

**Figure 1:**
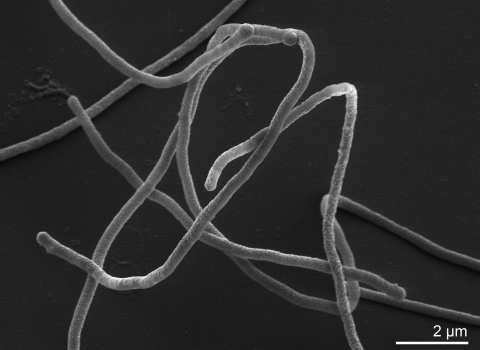
Scanning electron micrograph of *M. ruber* 21^T^

A detailed physiological description based on five strains has been given by Loginova *et al.* [5]. The cells are described as Gram-negative nonmotile rods that are 3 to 6 by 0.5 to 0.8 µm (Table 1), have rounded ends, and are nonsporeforming [5]. In potato-peptone-yeast extract broth incubated at 60°C, filamentous forms (20 to 40 µm in length) are observed along with shorter rods (Figure 1) [5]. No filamentous forms are observed after 16 h of incubation. *M. ruber* is obligately thermophilic [5]. On potato-peptone-yeast extract medium, the temperature range for growth is approx. 35-70°C, with an optimum temperature at 60°C (the generation time is then 60 min) [5]. A bright red intracellular carotenoid pigment is produced, which resembles retro-dehydro-γ-carotene (neo A, neo B) in its spectral properties [2]. The absorption spectra of acetone, methanol-acetone (l:l), and hexane extracts show three maxima at 455, 483, and 513 nm. The major carotenoid has since been identified as a 1‘-β-glucopyranosyl-3,4,3‘,4‘-tetradehydro-1‘,2‘-dihydro-β,ψ-caroten-2-one, with the glucose acetylated at position 6 [30]. One strain (strain INMI-a) contains a bright yellow pigment resembling neurosporaxanthine in its spectral properties [5], although it may well have been misidentified, since other species within the genus *Meiothermus* are yellow pigmented [8,9]. *M. ruber* is obligately aerobic [5]. It grows in minimal medium supplemented with 0.15% (wt/vol) peptone as an N source, 0.05% (wt/vol) yeast extract, and one of the following carbon sources at a concentration of 0.25% (wt/vol): D-glucose, sucrose, maltose, D-galactose, D-mannose, rhamnose, D-cellobiose, glycerol, D-mannitol, acetate, pyruvate, succinate, fumarate, or DL-malate (sodium salts). No growth occurs if the concentration of D-glucose in the medium is raised to 0.5% (wt/vol) [5]. Only moderate growth occurs when ammonium phosphate (0.1%, wt/vol) is substituted for peptone as the N source. No growth occurs in the control medium without a carbon source. No growth occurs on minimal medium supplemented with 0.25% (wt/vol) D-glucose, 0.05% (wt/vol) yeast extract and one of the following nitrogen sources at a concentration of 0.1% (wt/vol): L-alanine, glycine, L-asparagine, L-tyrosine, L-glutamate, ammonium sulfate, nitrate, or urea. Further lists of carbon source utilization, which differ in part from the above list, are published elsewhere [7,10-12]. Nitrates are not reduced and milk is not peptonized [5], but *M. ruber* strain 21^T^ is positive for catalase and oxidase [10]. The most comprehensive and updated list of physiological properties is probably given by Albuquerque et al [7].

**Table 1:** Classification and general features of *M. ruber* 21^T^ according to the MIGS recommendations [17]

**MIGS ID**	**Property**	**Term**	**Evidence code**
	Current classification	Domain *Bacteria*	TAS [18]
		Phylum *Deinococcus* -*Thermus*	TAS [1,3,19-23]
		Class *Deinococci*	TAS [24,25]
		Order *Thermales*	TAS [25,26]
		Family *Thermaceae*	TAS [25,27]
		Genus *Meiothermus*	TAS [1,7]
		Species *Meiothermus ruber*	TAS [5]
		Type strain 21	TAS [5]
	Gram stain	negative	TAS [5]
	Cell shape	rod	TAS [5]
	Motility	non motile	TAS [5]
	Sporulation	not reported	TAS [5]
	Temperature range	35°C–70°C	TAS [5]
	Optimum temperature	60°C	TAS [5]
	Salinity	growth with 1% NaCl	TAS [7]
MIGS-22	Oxygen requirement	obligately aerobic	TAS [5]
	Carbon source	a diverse set of sugars	TAS [5]
	Energy source	carbohydrates	TAS [5]
MIGS-6	Habitat	hot springs	TAS [5]
MIGS-15	Biotic relationship	free-living	TAS [5]
MIGS-14	Pathogenicity	not reported	
	Biosafety level	1	TAS [28]
	Isolation	hot spring	TAS [5]
MIGS-4	Geographic location	Kamchatka Peninsula, Russia	TAS [5]
MIGS-5	Sample collection time	1973 or before	TAS [2]
MIGS-4.1 MIGS-4.2	Latitude Longitude	unknown unknown	
MIGS-4.3	Depth	unknown	
MIGS-4.4	Altitude	unknown	

Evidence codes - IDA: Inferred from Direct Assay (first time in publication); TAS: Traceable Author Statement (i.e., a direct report exists in the literature); NAS: Non-traceable Author Statement (i.e., not directly observed for the living, isolated sample, but based on a generally accepted property for the species, or anecdotal evidence). These evidence codes are from of the Gene Ontology project [29]. If the evidence code is IDA, then the property was directly observed by one of the authors or an expert mentioned in the acknowledgements

**Figure 2:**
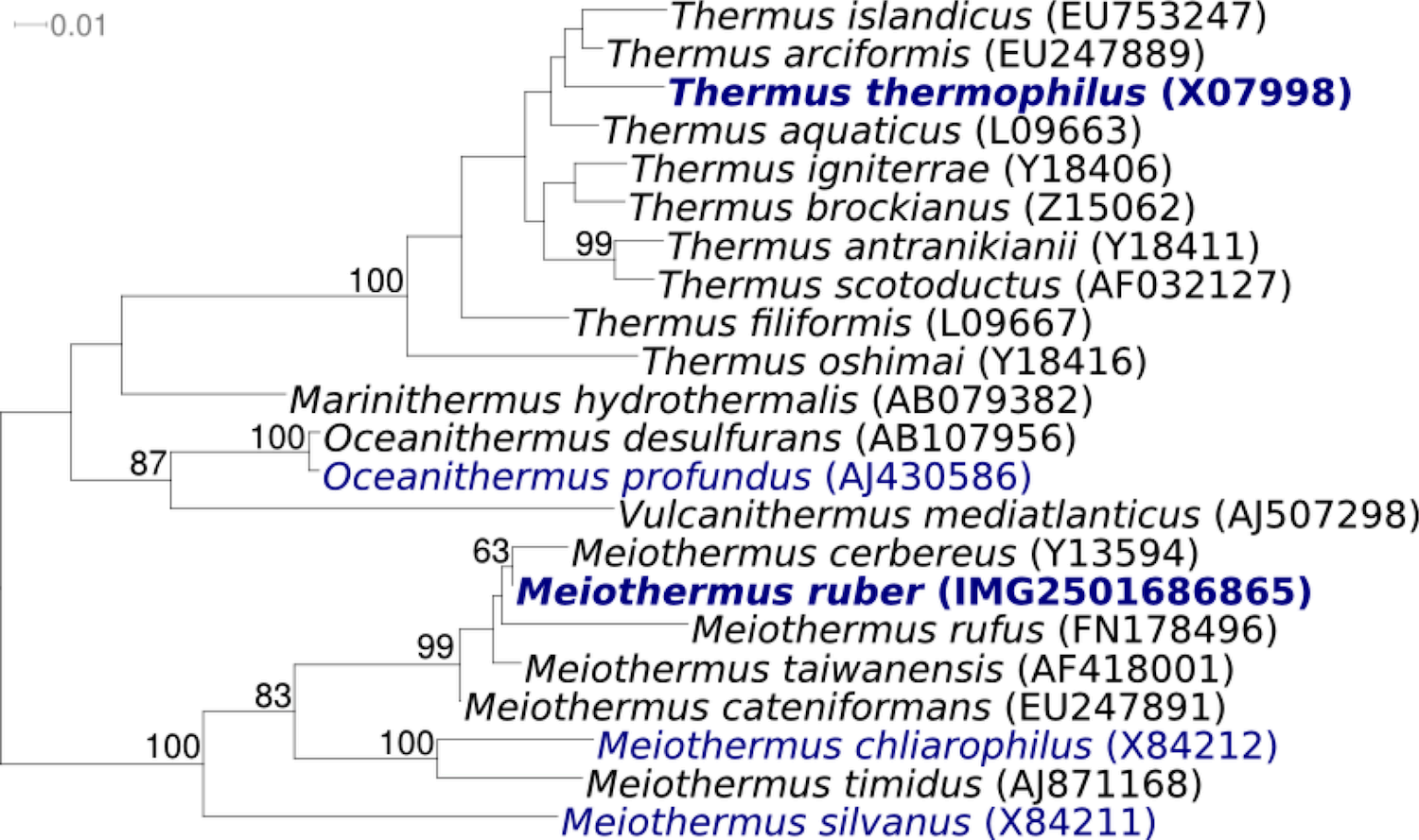
Phylogenetic tree highlighting the position of *M. ruber* 21^T^ relative to the type strains of the other species within the genus and to the type strains of the other species within the family *Thermaceae*. The trees were inferred from 1,403 aligned characters [31,32] of the 16S rRNA gene sequence under the maximum likelihood criterion [33] and rooted in accordance with the current taxonomy [34]. The branches are scaled in terms of the expected number of substitutions per site. Numbers above branches are support values from 1,000 bootstrap replicates [35] if larger than 60%. Lineages with type strain genome sequencing projects registered in GOLD [36] are shown in blue, published genomes in bold (*Thermus thermophilus*; AP008226).

Figure 2 shows the phylogenetic neighborhood of *M. ruber* 21^T^ in a 16S rRNA based tree. The sequences of the two 16S rRNA gene copies in the genome are identical and differ by only one nucleotide from the previously published sequence generated from ATCC 35948 (Z15059).

### Chemotaxonomy

Initial reports on the polar lipids of *M. ruber* indicated that they consist of two major glycolipids GL_1a_ (~ 42%) and GL_1b_ (~ 57%) and one major phospholipid PL_2_ (~ 93%), with small amounts of two other phospholipids PL_1_ and PL_3_ [37]. Detailed work indicates that in strains of *Thermus oshimai*, *T. thermophilus*, *M. ruber*, and *M. taiwanensis* the major phospholipid is a 2’-O-(1, 2-diacyl-*sn*-glycero-3-phospho) –3’-O-(α-N-acetyl-glucosaminyl)-N-glyceroyl alkylamine [38]. This compound is related to the major phosphoglycolipid reported from *Deinococcus radiodurans* [39] and can be considered to be unambiguous chemical markers for this major evolutionary lineage. The glycolipids are derivatives of a Glcp -> Glcp-> GalNAcyl -> Glcp -> diacyl glycerol [40]. Based on mass spectral data it appears that there may be three distinct derivatives, differing in the fatty acid amide linked to the gatactosamine [40]. These may be divided into one compound containing exclusively 2-hydroxylated fatty acids (mainly 2-OH iso-17:0) and a mixture of two compounds that cannot be fully resolved by thin layer chromatography carrying either 3-hydroxylated fatty acids or unsubstituted fatty acids. The basic glycolipid structure dihexosyl – N-acyl-hexosaminyl – hexosyl – diacylglycerol is a feature common to all members of the genera *Thermus* and *Meiothermus* examined to date. There is currently no evidence that members of the family *Thermaceae* (as currently defined) produce significant amounts of polar lipids containing only two aliphatic side chains. The consequences of having polar lipids containing three aliphatic side chains on membrane structure has yet to be examined. Such peculiarities also indicate the value of membrane composition in helping to unravel evolution at a cellular level. The major fatty acids of the polar lipids are iso-C15:0 (30-40%) and iso-C17:0 (13-17%), followed by anteiso-C_15:0_, C_16:0_, iso-C_16:0_, anteiso-C_17:0_, iso-C_17:0_-2OH, and, at least in some studies, iso-C_17:1_ *ω9c* (the values range from 3-10%). Other fatty acid values are below 2%, including 3-OH branched chain fatty acids. The values vary slightly between the different studies [7,9,11,12,37]. Detailed structural studies suggest that long chain diols may be present in small amounts, substituting for the 1-acyl-*sn*-glycerol [38]. Although not routinely reported the presence of alkylamines (amide linked to the glyceric acid of the major phospholipid) can be deduced from detailed structural studies of the major phospholipid [38]. Menaquinone 8 is the major respiratory quinone, although it is not clear which pathway is used for the synthesis of the naphthoquinone ring nucleus [41]. Ornithine is the major diamino acid of the peptidoglycan in the genus *Meiothermus* [1].

## Genome sequencing and annotation

### Genome project history

This organism was selected for sequencing on the basis of its phylogenetic position [42], and is part of the *** G****enomic* *** E****ncyclopedia of* *** B****acteria and* *** A****rchaea * project [43]. The genome project is deposited in the Genome OnLine Database [36] and the complete genome sequence is deposited in GenBank. Sequencing, finishing and annotation were performed by the DOE Joint Genome Institute (JGI). A summary of the project information is shown in Table 2.

**Table 2:** Genome sequencing project information

**MIGS ID**	**Property**	**Term**
MIGS-31	Finishing quality	Finished
MIGS-28	Libraries used	Three genomic libraries: one Sanger 8 kb pMCL200 library, one fosmide library and one 454 pyrosequence standard library
MIGS-29	Sequencing platforms	ABI3730, 454 Titanium
MIGS-31.2	Sequencing coverage	9.84× Sanger; 27.4× pyrosequence
MIGS-30	Assemblers	Newbler version 1.1.02.15, PGA
MIGS-32	Gene calling method	Prodigal 1.4, GenePRIMP
	INSDC ID	CP001743
	Genbank Date of Release	March 3, 2010
	GOLD ID	Gc01235
	NCBI project ID	28827
	Database: IMG-GEBA	2501651201
MIGS-13	Source material identifier	DSM 1279
	Project relevance	Tree of Life, GEBA

### Growth conditions and DNA isolation

*M. ruber* 21^T^, DSM 1279, was grown in DSMZ medium 256 (Nutrient Agar) [44] at 50°C. DNA was isolated from 0.5-1 g of cell paste using Qiagen Genomic 500 DNA Kit (Qiagen, Hilden, Germany) following the standard protocol as recommended by the manufacturer, with modification L for cell lysis as described in Wu *et al.* [43].

### Genome sequencing and assembly

The genome was sequenced using a combination of Sanger and 454 sequencing platforms. All general aspects of library construction and sequencing can be found at the JGI website (http://www.jgi.doe.gov/). Pyrosequencing reads were assembled using the Newbler assembler version 1.1.02.15 (Roche). Large Newbler contigs were broken into 3,428 overlap ping fragments of 1,000 bp and entered into assembly as pseudo-reads. The sequences were assigned quality scores based on Newbler consensus q-scores with modifications to account for overlap redundancy and adjust inflated q-scores. A hybrid 454/Sanger assembly was made using PGA assembler. Possible misassemblies were corrected and gaps between contgis were closed by primer walks off Sanger clones and bridging PCR fragments and by editing in Consed. A total of 431 Sanger finishing reads were produced to close gaps, to resolve repetitive regions, and to raise the quality of the finished sequence. Illumina reads were used to improve the final consensus quality using an in-house developed tool (the Polisher [45]). The error rate of the completed genome sequence is less than 1 in 100,000. Together, the combination of the Sanger and 454 sequencing platforms provided 37.24× coverage of the genome. The final assembly contains 30,479 Sanger reads and 371,362 pyrosequencing reads.

### Genome annotation

Genes were identified using Prodigal [46] as part of the Oak Ridge National Laboratory genome annotation pipeline, followed by a round of manual curation using the JGI GenePRIMP pipeline [47]. The predicted CDSs were translated and used to search the National Center for Biotechnology Information (NCBI) nonredundant database, UniProt, TIGRFam, Pfam, PRIAM, KEGG, COG, and InterPro databases. Additional gene prediction analysis and functional annotation was performed within the Integrated Microbial Genomes - Expert Review (IMG-ER) platform [48].

## Genome properties

**Figure 3:**
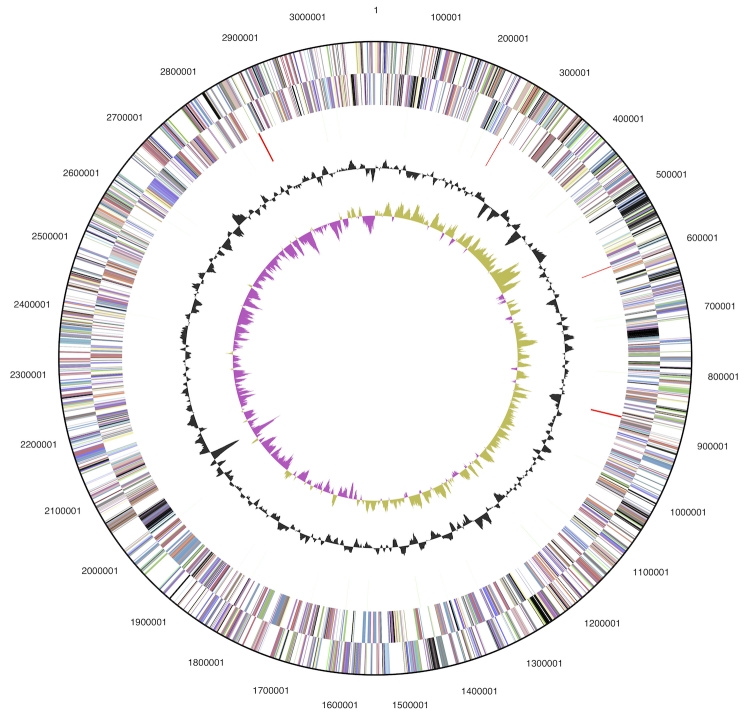
Graphical circular map of the genome. From outside to the center: Genes on forward strand (color by COG categories), Genes on reverse strand (color by COG categories), RNA genes (tRNAs green, rRNAs red, other RNAs black), GC content, GC skew.

The genome consists of a 3,097,457 bp long chromosome with a 63.4% GC content (Table 3 and Figure 3). Of the 3,105 genes predicted, 3,052 were protein-coding genes, and 53 RNAs; thirty eight pseudogenes were also identified. The majority of the protein-coding genes (71.8%) were assigned with a putative function while the remaining ones were annotated as hypothetical proteins. The distribution of genes into COGs functional categories is presented in Table 4.

**Table 3:** Genome Statistics

**Attribute**	**Value**	**% of Total**
Genome size (bp)	3,097,457	100.00%
DNA Coding region (bp)	2,807,535	90.64%
DNA G+C content (bp)	1,963,304	63.38%
Number of replicons	1	
Extrachromosomal elements	0	
Total genes	3,105	100.00%
RNA genes	53	1.71%
rRNA operons	2	
Protein-coding genes	3,052	98.29%
Pseudo genes	38	1.22%
Genes with function prediction	2,229	71.79%
Genes in paralog clusters	390	12.56%
Genes assigned to COGs	2,286	73.62%
Genes assigned Pfam domains	2,394	77.10%
Genes with signal peptides	1,079	34.75%
Genes with transmembrane helices	697	22.45%
CRISPR repeats	6	

**Table 4:** Number of genes associated with the general COG functional categories

**Code**	**value**	**%age**	**Description**
J	146	5.8	Translation, ribosomal structure and biogenesis
A	0	0.0	RNA processing and modification
K	131	5.2	Transcription
L	117	4.7	Replication, recombination and repair
B	2	0.1	Chromatin structure and dynamics
D	30	1.2	Cell cycle control, cell division, chromosome partitioning
Y	0	0.0	Nuclear structure
V	47	1.9	Defense mechanisms
T	103	4.1	Signal transduction mechanisms
M	114	4.5	Cell wall/membrane/envelope biogenesis
N	21	0.8	Cell motility
Z	1	0.0	Cytoskeleton
W	0	0.0	Extracellular structures
U	45	1.8	Intracellular trafficking and secretion, and vesicular transport
O	103	4.1	Posttranslational modification, protein turnover, chaperones
C	148	5.9	Energy production and conversion
G	190	7.6	Carbohydrate transport and metabolism
E	290	11.5	Amino acid transport and metabolism
F	81	3.2	Nucleotide transport and metabolism
H	102	4.1	Coenzyme transport and metabolism
I	95	3.8	Lipid transport and metabolism
P	139	5.5	Inorganic ion transport and metabolism
Q	61	2.4	Secondary metabolites biosynthesis, transport and catabolism
R	342	13.6	General function prediction only
S	208	8.3	Function unknown
-	819	26.4	Not in COGs
